# The ethics of sustainable genomic research in Africa

**DOI:** 10.1186/s13059-016-0914-3

**Published:** 2016-03-08

**Authors:** Michael Parker, Dominic P. Kwiatkowski

**Affiliations:** The Ethox Centre, Nuffield Department of Population Health, University of Oxford, Oxford, OX3 7LF UK; The Wellcome Trust Centre for Human Genetics, University of Oxford, Oxford, OX3 7BN UK; The Wellcome Trust Sanger Institute, Hinxton, Cambridge, CB10 1SA UK

## Abstract

Michael Parker and Dominic Kwiatkowski discuss important ethical considerations for sustainable genomics research in Africa.

## Why research is needed?

Diseases that affect the world’s poorest people remain woefully understudied relative to those affecting rich countries. The high burden of disease in sub-Saharan Africa provides a compelling reason for boosting medical research on the continent and bringing the latest technologies and analytical approaches to bear on the problem. The past decade has seen hopeful signs that the health needs of low-income countries are gaining increased attention among scientists and research funders, and one example of this is the growth of genomic research in Africa. It is an important development which, if combined with the collection of high-quality clinical and epidemiological data, will lead to an improved understanding of disease mechanisms and potentially powerful new tools for tackling the spread of drug resistance and emerging infections.

Genomic research in Africa presents a number of practical and ethical challenges. Some of the issues are common to any form of medical research conducted in low-income settings: these have been extensively discussed in the literature and are the subject of many, sometimes conflicting, guidelines [1]. However, the development of new forms of research collaboration and of technologies and methods associated with genomics are also leading to the parallel emergence of complex new ethical issues. This means that as well as following conventional ethical guidelines, genomics researchers need to consider a number of specific questions in order to achieve high ethical standards.

## Capacity building

There is much that is new about genomics, and arguably one of its most striking features is its dependence on international research collaboration. The growing emphasis on building global research partnerships is potentially an enormous opportunity for the growth of African science, but if the lessons of the past are to be learned and extractive research practices avoided, a key requirement is the training of a significant cohort of African scientists and clinicians with relevant expertise. This has implications for science education in African schools and universities but also for training at more senior levels for researchers and those running research institutions who may not have personal experience of genomics. There is also need for the development of a greater understanding of genomics among those who sit on research ethics or scientific review committees for whom genomics may be a relatively new concept, and who raise concerns because of lack of familiarity. Examples of large-scale genomics initiatives with a significant component of research capacity building include MalariaGEN, which brings together research groups in more than 30 malaria endemic countries in Africa and Asia; and the H3Africa initiative, which is funded jointly by the National Institutes of Health and the Wellcome Trust to promote African leadership of genomic research. It is encouraging that these and other capacity-building activities are starting to have an impact, as evidenced by the emergence of new African-led networks with a major focus on genetic research [2].

## Equitable research collaborations

The building of capacity is only part of what is needed for sustainable genomic research in Africa. Another key requirement is that international collaborative partnerships in genomics should be fair and equitable and that researchers in low-income settings are treated with respect as equal partners. It can be challenging to achieve equitable scientific partnerships when there are great disparities in resources and capacity, and this is all the more reason to strive for real evidence of capacity building and the creating of the conditions for equal partnership in the future [3, 4].

One area in which issues of fairness are of particular importance is data sharing. The sharing of genomic data across the international scientific community is an essential requirement for success in understanding of diseases affecting African populations, but it is vital for the sustainability of such research that the ground rules for data sharing should be equitable. The challenge is to create a level playing field between data producers and data users that respects the legitimate interests of African researchers who have generated samples and data. It is important to recognize that occasionally there may be tensions between the potential short-term benefits of rapid data release and the longer-term benefits of fostering sustainable scientific research in Africa. In some cases there may be good reasons — grounded both in the importance of fairness and sustainable science — for giving preferential data access for a limited time to groups based in low-income settings [5]. This may also be important for the promotion of public trust and confidence.

## Public trust

Of paramount importance to the sustainability of genomics research in Africa is that publics, research participants, and frontline research workers should have good grounds for placing their trust in the people and institutions conducting genomic research. A recent study on attitudes to data-sharing in low-income settings identified a great deal of public support for sharing of data but also identified trust, the minimizing of harm, and fairness and reciprocity as key requirements for such support [6]. Concerns about the sharing and uses of research samples and data across international boundaries are not unique to low-income countries, but they can take on particular moral and political resonance in such contexts because of enduring global inequalities and worries about the potential for neocolonial, extractive research practices. Such worries may be compounded by the newness and scale of genomics and by the notion, which researchers are naturally keen to foster, that these are particularly valuable materials and data. If the benefits of genomic research for Africa are to be realised, then careful attention needs to be paid to the achievement of well-founded and sustainable public trust and confidence in the conduct of genomic research and the international collaborations essential to its success. An important role in the building of public trust and hence of sustainable scientific collaboration, particularly in the context of persisting global health and other inequality and fragile trust, is likely to be played by the clear articulation of the “social contract” for genomics research in Africa and the establishment of widely respected models of good practice [7].

## High ethical standards

We have chosen to highlight issues related to capacity building, fairness in research collaboration, and public trust because it is crucial, in our view, that greater attention is paid to these. However, it is also important to continue to identify and address the day-to-day practical ethical issues that genomics research in Africa presents on the ground in real world settings.

An important example is the achievement of valid consent. Although the requirements for the achievement of valid consent have been a central concern of research ethics throughout its history, genomics presents important new problems and asks difficult questions about the role of consent and the scope of the ethical protections it is able to provide. Valid consent is usually understood to be consent that is informed, voluntary, and competent. In the context of genomics, the meeting of each of these requirements presents important practical problems. Perhaps the greatest difficulties are those related to information and understanding: key concepts in genomic research can be difficult to explain as are many of the practices essential to genomics such as data-sharing and the use of data in future as yet undetermined research by researchers, often at great distance from the context in which the data were obtained. Such uses of future research may have the potential to make an important contribution to the understanding of the diseases affecting people living in Africa; but what model of consent is most appropriate for this? Important work is currently being done on the development of models of good consenting practice suited to the recruitment of participants from low-income settings in genomic research [8]. In high-income countries such questions are starting to be resolved in favor of approaches combining high-quality broad consent with independent governance of data access. Research on attitudes to data sharing in low-income settings and consultation in the context of initiatives such as H3Africa suggest that such models may be also acceptable here, but only where they are complemented by additional protections such as material transfer agreements, data access agreements, and independent and trusted forms of governance with robust local involvement [6].

## Conclusion

There are good prima facie reasons for supporting the growth of genomic research in low-income settings because of its potential to improve understanding of the diseases affecting the world’s poorest people. Such research is only sustainable and ethical in the context of fair research collaborations, genuine commitment to the building of research capacity, and effective and trusted governance informed by awareness of the expectations of those living in the settings from which data and samples were obtained about what constitutes good research practice. The achievement of high ethical standards, for example in regard to the achievement of valid consent, requires further work at national and international levels on the establishment of widely shared models of good research practice. The focus must be on the achievement of best ethical practice in real-world settings. This requires the development of innovative models for the embedding of ethics in the day-to-day conduct of large-scale genomic research collaborations involving partners from high-income and low-income settings.
